# Isolation and antimicrobial resistance patterns of Methicillin‐resistant *Staphylococcus aureus* from raw cow's milk in dairy farms of Wolaita Sodo Town, Southwest, Ethiopia

**DOI:** 10.1002/fsn3.4121

**Published:** 2024-03-25

**Authors:** Hailehizeb Tegegne, Eyoel Ejigu, Dese Woldegiorgis, Azeb Mengistu

**Affiliations:** ^1^ College of Agriculture and Environmental Science Debre Tabor University Debre Tabor Ethiopia; ^2^ College of Agriculture and Veterinary Medicine, Jimma University Jimma Ethiopia

**Keywords:** dairy farms, milk, MRSA, Wolaita Sodo

## Abstract

*Staphylococcus aureus* is a pathogenic bacterium‐contaminating milk and milk products causing food poisoning primarily due to its enterotoxins. A cross‐sectional study was conducted from November 2021 to June 2022 in Wolaita Sodo Town, to detect Methicillin‐resistant *Staphylococcus aureus* (MRSA) in raw cow's milk and assess their resistance levels to different antimicrobials. Purposive sampling was used to select 34 dairy farms. Accordingly, 419 raw milk samples from the farm and collection centers were collected. Isolates of *S. aureus* showing resistance to Cefoxitin were classified as MRSA. From the total 419 samples, 22.19% (93/419) were contaminated with *S. aureus* in dairy cows. The prevalence of *S. aureus* in raw milk, bulk milk from the farm, and bulk tank milk from the collection centers was 16.9%, 2.1%, and 3.1%, respectively. The risk of *S. aureus* contamination in dairy farm owners and milkers who do not take food safety training was 5.303 times higher than the risk of *S. aureus* contamination in dairy farm owners and milkers who take food safety training. The risk of *S. aureus* contamination in dairy farms kept under poor management system was 7.34 times more than that of dairy farms kept under good management. The cefoxitin disk diffusion method was used to detect MRSA, 57.14% being resistant to Cefoxitin in total while approximately 87.5% were sensitive to Sulfamethoxazole‐trimethoprim and Gentamycin, while Erythromycin registered 75%, Ciprofloxacin 62.5%, Chloramphenicol 62.5%, and Tetracycline 25%. This study revealed that MRSA exhibited a notable multidrug resistance pattern, encompassing resistance to multiple drugs, with a prevalence of 75%. Significantly, the hands of milkers and the milking containers emerged as pivotal sources of contamination. This underscores the crucial importance of maintaining stringent hygienic practices during the milking process, with particular emphasis on thorough cleaning and decontamination of utensils.

## Graphical abstract

Out of the total 419 milk samples, 22.19% (93/419) were contaminated with *Staphylococcus aureus* in dairy cows. And the antibiotic resistance profile of *S. aureus* showed 100% resistance to Penicillin, 95% resistance to Cefoxitin, and 90% resistance to Tetracycline. Coversely, *S. aureus* showed 95% sensetivity to Sulfamethoxazole‐trimethoprim, 92.5% to Gentamycin, and 92% to Chloramphenicol. This study revealed that Methicillin‐resistant *Staphylococcus aureus* (MRSA) exhibited a notable multidrug resistance pattern, encompassing resistance to multiple drugs, with a prevalence of 75%.

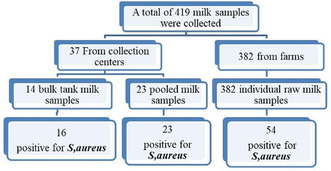

## 1 INTRODUCTION

### 1.1 Background and justification

In less developed nations, the primary reason for illness and fatalities is attributed to contaminations transmitted through food. These illnesses stem from shifts in dietary habits, large‐scale food service operations, inappropriate storage practices, and insufficient adherence to hygiene protocols. Consequently, these factors contribute to 600 million instances of illness and 33 million deaths globally (Mekibib et al., 2010). *S. aureus* case fatality rates are 0.03% (Mekibib et al., 2010) in poor countries like Ethiopia, where milk production and other dairy products are mostly prepared under unclean situations and the consumption of raw milk and under cooked milk and milk products is common.

Pathogenic microorganisms in milk may be sourced from the cow itself, the human hand, or the environment (Abera et al., 2013). Milk and its derivatives are prone to microbial contamination due to their nutrient‐rich composition, creating an optimal environment for the proliferation of various microorganisms. Contamination can occur through multiple origins, such as *Staphylococcus aureus* contamination in the mammary gland or inadequate hygienic practices like coughing, sneezing, and insufficient handwashing during the handling of milk, milk storage, and milking processes. In this context, human‐related factors play a great role in contamination, as the bacteria; in this case, the bacteria inhabit the nasal passages of humans (Abunna et al., 2016).

*Staphylococcus aureus* is an adaptable pathogen of animals and humans that causes a wide array of diseases. It occurs in different locations on dairy farms and many studies recommended that the transmission of *S. aureus* between humans and cows is possible. Mostly transmission occurs at the milking time through contaminated milking machines, clothes, and the hands of milkers or machine operators (Radostis et al., 2007). Dairy farms serve as significant repositories for *S. aureus* pathogens. This *S. aureus* can move in the farm via infected and noninfected cows, farm workers, and the environment, and there is a risk that it may sporadically transfer to bulk tank milk, presenting a public health concern for consumers (Haran et al., 2012). *S. aureus* is a leading pathogen causing many serious diseases in dairy and healthcare surroundings (Mekibib et al., 2010). In the last few decades, Staphylococcal food poisoning has been reported as the third cause of foodborne illnesses in the world (Mekibib et al., 2010).

Additionally, the presence of antibiotic‐resistant strains of *S. aureus* poses a significant challenge for both veterinary and healthcare professionals, and dairy cattle producers, due to its adverse impact on treatment efficacy. This bacterium produces various exoproteins that enhance its capacity to invade the mammary gland, including membrane‐damaging hemolysin toxins (alpha, beta, gamma, and delta), and leukocidin. Among these, alpha and beta hemolysins play a crucial role in the development of intramammary contaminations. Staphylococcal protein A, a membrane‐bound exoprotein, is particularly notable for its well‐known capability to bind to the fragment crystallizable (Fc) region of immunoglobulins found in most mammalian species (Radostis et al., 2007).

Several studies in Ethiopia indicated that *S. aureus* is associated with approximately 40% of mastitic cases in cows, as reported by many authors (Abera et al., 2013; Dego & Tarake, 2003; Koye et al., 2008; Shibeshi et al., 2002). In a study conducted by Sori et al. (2005), the prevalence of *S. aureus* causing mastitis was found to be 44%, with *Staphylococcus* epidermidis accounting for 14.93% of cases. Given the high prevalence of subclinical mastitis in Ethiopia, the potential public health risks of zoonotic pathogens become a significant concern, particularly with the common practice of consuming raw milk from apparently healthy cows (CSA, 2001).

Reports highlight increasing resistance among *S. aureus* isolates to multiple antibiotics. Despite the availability of various antibiotics, cases of *S. aureus* mastitis often exhibit poor responsiveness to drug treatments, leading to substantial losses in the global dairy industry (Normanno et al., 2007). The notable capacity of *S. aureus* to develop drug resistance has resulted in the emergence of Methicillin‐resistant *S. aureus* (MRSA). Traditionally, Penicillin and its derivatives, including Methicillin, were used to treat *S. aureus* contaminations. However, resistance has arisen due to an alternative Penicillin‐binding protein, PBP2a, encoded by the *mecA* gene located in the Staphylococcal cassette chromosome (*SCCmec*) mobile genetic element (Vanderhaeghen et al., 2010).

Historically, *Methicillin‐resistant Staphylococcus aureus* (*MRSA*) was predominantly inherent in nosocomial contaminations, categorized as Healthcare‐Associated MRSA (HA‐MRSA) until the 1990s (Rayner & Munckhof, 2005). MRSA is further classified into three groups based on epidemiological and genetic characteristics: Hospital‐acquired (HA‐MRSA), Community‐associated (CA‐MRSA), and Livestock‐associated (LA‐MRSA).

The various groups exhibit differences in their susceptibility to antibiotics, the location and size of the chromosomal cassette (*SCCmec*), and the presence of the Panton–Valentine leukocidin gene (PVL) (Joshi et al., 2014). The inappropriate use of antimicrobials in dairy farms, whether for therapeutic or for preventive purposes, has the potential to foster resistance mechanisms in *S. aureus*, hastening the emergence of multidrug resistant strains like MRSA. And, due to lateral gene transfer, the prevalence of antibiotic‐resistant genes in the *Staphylococcus* species is a major problem, as these resistant bacterial strains can be carried from animals to humans (Lubna et al., 2023). Inadequate awareness and unhygienic practices, such as the consumption of raw milk or insufficient use of personal protective equipment (PPE) and handwashing, may elevate the risk of occupational exposure to MRSA. The threat of *MRSA* associated with dairy farms could extend to public health if these strains gain entry into milk or spread environmentally through contaminated air, water, or manure to populations residing in proximity to dairy farms (Elmonir et al., 2019). While comprehensive studies on antimicrobial resistance (AMR) in Ethiopia are lacking, available reports strongly suggest that it is an emerging issue deserving attention as a public health concern (DACA, 2009; Gebre‐Sealsssie, 2007). Although there is limited information on the public health impact of *Methicillin‐resistant Staphylococcus aureus* (*MRSA*) associated with food‐producing animals in Ethiopia, various reports in the veterinary field highlight the common occurrence of *S. aureus* resistance to antimicrobials in different regions of the country (Abera et al., 2013; Daka et al., 2012; Sori et al., 2011).

Asmelash Tassew et al. (2016) reveal that approximately 47.2% of *S. aureus* from milk exhibit resistance to Cefoxitin, with 45.3% demonstrating resistance to four antibiotic disks (penicillin G, ampicillin, cefoxitin, and erythromycin). However, there are limited data on the antimicrobial resistance patterns of *S. aureus* and *MRSA* in raw milk specifically from Wolaita Sodo Town. Therefore, this study aims to assess the antibiotic response profile of *MRSA* to understand the extent of resistance in the study area.

## 2 MATERIALS AND METHODS

### 2.1 Study area

The study was conducted from November 2021 to June 2022 in and around Wolaita Soddo town, located in Southern Ethiopia. The Wolaita zone is positioned approximately 329 km south of Addis Ababa, ranging in altitude from 700 to 2950 m above sea level and covering a total land area of 4537.5 square kilometers. Geographically, it is situated between 6°4′ N to 7°1′ N and 37°4′ E to 38°2′ E. The region experiences a mean annual rainfall between 450 and 1446 mm, with a bimodal distribution featuring a major rainy season from June to September and a shorter rainy season from February to April. The average annual temperatures in the area are recorded as 34.12°C max and 11.4°C min, respectively. The prevalent agricultural system is characterized by mixed crop–livestock production. The estimated livestock population in the Wolaita zone includes 886,242 bovines, 117,274 ovines, 99,817 caprines, 41,603 equines, and 442,428 poultry. The zone comprises 12 Woredas (Districts).

### 2.2 Study population

This study focused on milk samples collected from private, commercial, and governmental dairy farms from Wolaita Sodo Town. This study had taken up a commitment to scientific inquiry and dedication to the ethical treatment of animals involved in our research endeavors. We prioritized the well‐being of animals involved in our research. All measures were taken to ensure that animals are treated with the utmost care, respect, and consideration for their natural behaviors and needs. Housing, handling, and overall conditions were optimized to promote their physical and psychological welfare.

### 2.3 Study design and sample type

A cross‐sectional study was conducted from November 2021 to June 2022 to analyze, identify, and assess the antimicrobial susceptibility patterns of MRSA in dairy milks. The research involved obtaining samples from various sources, including raw milk, farm pooled milk, collection center pooled milk, milkers of dairy cows, and materials utilized in the milking process. Sampling days were randomly selected, and every sampled farm was visited twice during the study period. To facilitate the sampling process, a cooperation letter was dispatched to the authorities at Wolaita Sodo's livestock resource development office in advance, and an animal health technician was designated to assist with the sampling at each dairy farm.

### 2.4 Sampling technique and sample size determination

The sample size was determined using Thrusfield (2018). Accordingly, a 95% confidence interval, 5% absolute precision, and an expected prevalence of 47%, as previously reported by Mekibib et al. (2010) were used.

$$N=Z^{2*}\;{P_{\exp}}^{*}\;\left(1-P_{\exp}\right)/D^{2},$$

Where *Z* = 1.96, *N* = sample size, *P*_exp_ = expected prevalence, and *D* = absolute precision. Aligned with these specified criteria, an extensive collection effort resulted in the collection of a total of 419 milk samples originating from a network of 34 diverse dairy farms. This encompassed the gathering of 382 individual raw milk samples, meticulously obtained from distinct cows within various farms, alongside 14 bulk tank milk samples sourced from different dairy establishments. Additionally, 23 pooled milk samples were meticulously acquired from designated collection centers, ensuring a nuanced and representative selection for thorough analysis.

### 2.5 Sample collection and transportation

Approximately 382 milk samples were taken from lactating dairy cows in adherence to a previous procedure. The collection involved using sterile cotton swabs, screw‐capped test tubes, peptone water, and wooden sticks to obtain 10–15 mL of milk from each sample. A composite sample of pooled milk from the dairy farm was taken postmilking, combining the milk from all cows in a designated container. Similarly, samples from pooled milk at collection centers were gathered after consolidating milk sourced from various locations, with subsequent mixing and careful collection posthomogenization. The collection times, either early morning or late afternoon, were determined by the convenience of the milkers, collection centers, and farm owners. The coded samples were then taken to the microbiology laboratory at the Wolaita Sodo University Teaching Referral Hospital for processing.

### 2.6 Questionnaire survey

Information on potential sources of *coagulase positive staphylococci* (CPS) contamination in milk was gathered through personal observation and a structured questionnaire. The study examined various factors contributing to contamination, including the cleanliness of the utensils and milking conditions, the health status and cleanness of milking cows’ udders, the cleanliness of milk handlers, and the hygiene of milking utensils. Special attention was given to the practices and utensils used for milking. Additionally, the overall hygiene of the housing facilities was evaluated. Face‐to‐face interviews were conducted with one representative milker from each of the 34 dairy farms to complete the questionnaire.

### 2.7 Laboratory examination

#### 2.7.1 Culturing and biochemical tests

A loopful of milk was streaked onto 5% sheep blood agar loops and incubated at 37°C for 24 h. Colonies were characterized based on Gram staining, cellular morphology, and hemolytic patterns. Selected colonies were cultured on mannitol salt agar, followed by subculturing on nutrient broth and agar plates at 37°C for 24–48 h to obtain pure cultures. Pure isolates were preserved at 4°C for subsequent biochemical tests based on Quinn et al. (2002) protocols. Identification of *coagulase positive staphylococci* involved catalase and tube coagulase tests. Colonies exhibiting hemolysis‐positive, Gram‐positive, catalase‐positive, and coagulase‐positive traits were recognized as *coagulase positive staphylococci*.

#### 2.7.2 Antimicrobial susceptibility testing

The *coagulase positive staphylococci* isolates were submitted to determine their antimicrobial susceptibility testing through the disk diffusion method. The assessment included the use of the following antibiotics: Cefoxitin (30 μg), Penicillin G (10 μg), Tetracycline (30 μg), Gentamycin (10 μg), Chloramphenicol (30 μg), Erythromycin (15 μg), Ciprofloxacin (5 μg), and Sulfamethoxazole‐trimethoprim (25 μg). To begin with, 3–5 colonies from a pure culture were transferred into test tubes containing sterile saline, thoroughly mixed, and the suspension's turbidity was adjusted by comparing it with a 0.5 McFarland standard.

Subsequently, a Muller–Hinton agar plate was made ready, and a sterile cotton swab was immersed in the suspension, which was then spread across the surface of the Muller–Hinton agar plate. Antibiotic disks were placed on the agar plate using sterile forceps and pressed gently to ensure complete contact with the agar surface. After 24 h of incubation at 37°C under aerobic conditions, the plates were examined.

The classification of isolates as susceptible, intermediate, or resistant for each antibiotic followed the guidelines of the Clinical Laboratory Standards Institute (CLSI). This classification was determined by measuring the zone of inhibition around the antibiotic disk, and the diameter of this zone was measured in millimeters using a ruler (CLSI, 2018), by the manufacturer's instructions.

#### 2.7.3 Identification of MRSA

For the identification of MRSA, a cefoxitin (30 μg) disk was used. It is stated in the absence of Methicillin, the best alternative is to use Cefoxitin for MRSA identification (NCCLS, 2011). *S. aureus* isolates exhibiting resistance to Cefoxitin were identified as MRSA as suggested (CLSI, 2018).

### 2.8 Data management and statistical analysis

The data were encoded and inputted into a Microsoft Excel 2010 spreadsheet, where they underwent a thorough accuracy check. Following validation, the data were transferred and processed using SPSS version 20 software for analysis. Descriptive analysis was employed to depict the results of the proportion analysis, where the proportion was determined by assessing the number of samples identified as positive for MRSA isolation from *coagulase positive staphylococci* isolates.

## 3 RESULTS

### 3.1 Prevalence of *coagulase positive staphylococci* (CPS)

In pursuit of understanding the microbial landscape within dairy production, the study investigation aimed to analyze 419 milk samples collected from 34 diverse dairy farms. With a focus on assessing the prevalence of *S. aureus*, our study set out to explore potential contamination in various types of milk, including raw milk, pooled milk from the farm, and bulk tank milk from collection centers. Out of the 419 samples analyzed, 22.19% (93 out of 419) exhibited *coagulase positive staphylococci* contamination in dairy cows. The prevalence of *S. aureus* was found to be 16.9% in raw milk, 2.1% in pooled milk from the dairy farm, and 3.1% in bulk tank milk from the collection center (Table 1).

**TABLE 1:** Prevalence of *coagulase positive staphylococci*. in different milk samples.

Sample type	No of tested	No of positive	Prevalence	Mean	Std. deviation
Raw milk	382	71	16.9	1.81	0.390
Pooled milk from the farm	14	9	2.1	1.36	0.497
Pooled milk from collection center	23	13	3.1	1.43	0.507
Total	419	93	22.19	1.81	0.390

A univariate logistic regression analysis was conducted on distinct risk factors using independent samples. The results indicated significant associations (*p* < .05) between *coagulase positive staphylococci* contamination risk and various factors, including the dairy farm's management system, the breeds of dairy cattle (Holstein Friesian (HF) cross and Jersey), and the production system, as depicted in the following results (Table 2).

**TABLE 2:** Association of potential risk factors with *S. aureus* contamination.

	Variable	SE	COR	95% CI	*p*‐Value
Sanitation	Good	Ref	Ref	Ref	Ref
	Moderate	0.271	0.728	0.428–1.238	.241
	Poor	0.277	1.263	0.735–2.173	.398
Breed	HF cross	Ref	Ref	Ref	Ref
	Jersey	0.220	0.444	0.288–1.684	.067
Management	Good	Ref	Ref	Ref	Ref
	Medium	.289	0.774	0.440–1.362	.374
	Poor	0.311	0.149	0.081–0.274	.000
Production	Semi‐intensive	Ref	Ref	Ref	Ref
	Extensive	0.208	2.118	1.308–3.187	.000
Farm hygiene	Moderate	Ref	Ref	Ref	Ref
	Poor	0.212	0.549	0.362–0.832	.005
Farm size	Small	Ref	Ref	Ref	Ref
	Large	0.209	1.690	1.121–2.548	.012
Food safety training	Yes	Ref	Ref	Ref	Ref
	No	0.390	5.305	2.471–11.389	.000

Abbreviations: COR, crude odd ratio; Ref, reference.

### 3.2 Risk factor analysis

In this study, various factors were analyzed using multivariable logistic regression to assess their impact on the risk of *coagulase positive staphylococci* contamination. The factors included sanitation, breed, production system, management system, farm hygiene, farm size, and food safety training. Among these, food safety training, farm size, management, farm hygiene, and production system were found to be statistically significant (*p* < .05) in their association with the risk of *coagulase positive staphylococci* contamination. However, breed and sanitation did not show a statistically significant association with the risk of *coagulase positive staphylococci* contamination.

Specifically, food safety training had a highly significant impact on the risk of *S. aureus* contamination in dairy cows, with a *p*‐value of .001. This study indicated a substantial protective effect associated with food safety training among dairy farm owners and milkers. Those who took food safety training were identified to be at a remarkable 5.303 times lower risk of *Staphylococcus aureus* (*S. aureus*) contamination compared to their counterparts who did not receive such training. This compelling finding prompts a deeper exploration into the potential mechanisms underlying the observed difference. Those individuals with food safety training could exhibit enhanced awareness and adherence to hygienic practices, thereby reducing the likelihood of *coagulase positive staphylococci* contamination. The training might empower dairy farm owners and milkers to implement more effective preventive measures, such as stringent sanitation protocols and proper handling procedures.

The management system also had a significant association with the risk of *coagulase positive staphylococci* contamination in dairy cows, with a *p*‐value of .001. Dairy farms with poor management systems had a 7.34 times higher risk of *coagulase positive staphylococci* contamination compared to those with good management systems. Similarly, dairy farms with medium management systems had a 5.1 times higher risk of contamination compared to those with good management systems.

The production system was significantly associated with the risk of *coagulase positive staphylococci* contamination in dairy cows, with a *p*‐value of .032. Dairy cows kept under extensive production systems had a 3.2% lower risk of *coagulase positive staphylococci* contamination compared to those under semi‐intensive production systems.

Farm hygiene also had a significant impact on the risk of *S. aureus* contamination, with a *p*‐value of .001. Dairy farms with poor hygiene status had a 2.88 times higher risk of *coagulase positive staphylococci* contamination compared to those with moderate farm hygiene (Table 3).

**TABLE 3:** Association *coagulase positive staphylococci*. contamination with potential risk factors.

Variable		*N*	Prevalence	AOR	95% CI	*p*‐Value
Sanitation	Good	159	37.9	Ref	Ref	Ref
	Moderate	167	39.9	0.484	0.245–0.958	.037
	Poor	93	22.2	1.091	0.561–2.123	.798
Breed	HF cross	166	39.6	Ref	Ref	Ref
	Jersey	253	60.4	2.332	0.373–3.963	.85
Management	Good	102	24.3	Ref	Ref	Ref
	Medium	113	27.0	5.053	2.804–9.105	.000
	Poor	204	48.7	7.34	3.579–15.035	.000
Production	Semi‐intensive	204	48.7	Ref	Ref	Ref
	Extensive	215	51.3	0.589	0.303–0.956	.032
Farm hygiene	Moderate	180	43.0	Ref	Ref	Ref
	Poor	239	57.0	2.8828	1.711–4.674	.000
Farm size	Small	232	55.4	Ref	Ref	Ref
	Large	187	44.6	1.073	0.649–1.774	.783
Food safety training	Yes	35	8.4	Ref	Ref	Ref
	No	384	91.6	5.305	2.471–11.389	.000

Abbreviations: AOR, adjusted odds ratio; *N*, number; Ref, reference.

### 3.3 Antimicrobial susceptibility test

An antimicrobial susceptibility test was done on all *S. aureus* isolates. The results indicated that all MRSA isolates demonstrated resistance to Penicillin (100%). Additionally, *S. aureus* isolates exhibited a high resistance rate to mostly used antimicrobial drugs in both veterinary and human health. Specifically, *S. aureus* displayed significant “in vitro resistance” to antibiotics, such as Cefoxin (95%), Tetracycline (95%), Penicillin (100%), and Ciprofloxacin (62.5%). Table 4 provides details on the number of isolates sensitive to various antibiotics. A majority of MRSA isolates were sensitive to Sulfamethoxazole‐trimethoprim (95%) and Gentamycin (92.5%), followed by Erythromycin (90%) and Chloramphenicol (92.5%), while Ciprofloxacin exhibited a sensitivity of 62.5%. Notably, Tetracycline demonstrated the lowest sensitivity at 25% (Table 4). Based on this investigation, 75% of *Methicillin‐resistant Staphylococcus aureus* (*MRSA*) isolates were found to concern the profile of multidrug resistance (MDR). This finding underscores the complex and difficult nature of the antimicrobial challenges posed by a significant majority of the MRSA strains examined in the study (Table 5).

**TABLE 4:** Antibiotic susceptibility pattern of MRSA isolates (*n* = 40).

Antibiotics used	Antibiotic susceptibility profile		
	No. sensitive (%)	No. intermediate (%)	No. resistant (%)
Erythromycin	36 (90%)	–	2 (5%)
Gentamycin	37 (92.5%)	–	1 (25%)
Ciprofloxacin	25 (62.5%)	1 (25%)	25 (62.5%)
Cefoxitin	–	–	38 (95%)
Tetracycline	12 (30%)	–	36 (90%)
Penicillin	–	–	40 (100%)
Chloramphenicol	35 (92.5%)	1 (25%)	2 (5%)
Sulfamethoxazole‐trimethoprim	38 (95%)	–	1 (25%)

**TABLE 5:** Hygienic practices by milking personnel.

Variables	Milking personnel (*N* = 50)
Hand washing before milking	Yes 49 (98%)
	No 1 (2%)
Hand washing between milking processes	Yes 10 (20%)
	No 40 (80%)
Udder washing before milking	Yes 28 (56%)
	No 22 (44%)
Udder dried after washing	Yes 26 (52%)
	No 24 (48%)
Use of separate drying towel for udder	Yes 2 (4%)
	No 48 (96%)
Use of detergent for milk container	Yes 50 (100%)
	No 0 (0%)
Housing general hygiene	Yes 25 (50%)
	No 25 (50%)

### 3.4 Questionnaire survey

In this study, 50 participants took part. Among them, 56% and 52% of individuals responsible for milking were engaged in udder washing and drying activities before milking, respectively. Notably, 96% did not employ separate drying towels for the udder. Conversely, 80% did not adhere to the practice of washing their hands between milking sessions, although 98% consistently washed their hands before milking. It is noteworthy that all individuals involved in milking followed the practice of cleaning milking equipment with detergents before the milking process.

## 4 DISCUSSION

In this investigation, 22.19% (93 out of 419) of the *coagulase positive staphylococci* were identified from a total of 419 samples collected from both small‐ and large‐scale dairy farms in Wolaita Sodo Town. These results corroborate those of Abera et al. (2012) and Joshi et al. (2014), who reported 24.1% of *coagulase positive staphylococci* in Shashemene, Ethiopia, and 23% of *S. aureus* in Pokhara, Nepal, respectively. However, they differ from the results of Riva et al. (2015), who observed 9.1% of *coagulase positive staphylococci* from bovine raw milk in Italy. Furthermore, they surpass the findings of Suleiman et al. (2012) in Nigeria and Mekonnen Hailemariam et al. (2005) in Ethiopia, both of whom isolated 8% *of coagulase positive staphylococci* from bovine raw milk.

The difference in the prevalence of *coagulase positive staphylococci* across different studies may be attributed to variations in environmental conditions, management systems, sample sizes, and hygienic practices employed in farms and milk collection centers. Additionally, differences in animal production systems among countries could contribute to these variations. It is worth noting that *coagulase positive staphylococci* are likely communicable pathogens that can be transmitted among cows or from individual to individual through contact with animals during unclean milking activities, as suggested by Rowe (1999).

In this investigation, 57.5% (23 out of 40) of the isolates were identified as MRSA from various origins. These findings agree with those of Elemo et al. (2017), who observed a similar prevalence of 58.1% in and around Asella, Ethiopia. Additionally, it is consistent with Daka et al.'s (2012) discovery of 60.3% MRSA among *S. aureus* isolates in Ethiopia and Locatteli et al.'s (2016) findings of 60% MRSA in Italy. However, it is noteworthy that our findings are lower than those reported by Tassew et al. (2017), who identified 75.7% MRSA isolates in Bahir Dar and 74.11% MRSA isolates in and around Assosa, Ethiopia, respectively.

The high prevalence of MRSA in dairy cattle could be attributed to the extensive use of antibiotics for managing bacterial contaminations, such as bovine mastitis. Indiscriminate antibiotic use may contribute to the existence of multidrug‐resistant bacterial strains and an increased likelihood of drug residues in milk (Aklilu & Chia, 2020). The substantial presence of these bacteria in milk and its products may be linked to inadequate hygienic practices, including unclean hands of milkers and unhygienic milking equipment and environments. Milk collected directly from farms or markets is susceptible to contamination under unsanitary conditions.

In a more recent study from Malaysia by Aklilu and Chia (2020), 38% of the 44 *S. aureus* isolates were confirmed as MRSA using the cefoxitin disk diffusion test and *mecA* detection. This prevalence is lower than that observed in the present study. The variance in prevalence outlined by these authors may be attributed to the use of the PCR (polymerase chain reaction) method for MRSA detection, which has higher sensitivity and specificity, resulting in a lower detection rate of *S. aureus* compared to the culture method (Khakpoor et al., 2011).

Regarding the proportion of *S. aureus*, our study found rates of 16.9%, 2.1%, and 3.1% in raw milk, pooled from the farm, and pooled milk from the collection center, respectively. These results are consistent with the findings of Regasa et al. (2019) and Mekuria et al. (2013) in the Oromia region. However, they differ from the findings in South‐West Uganda, Iran, and Annand, Gujarat, where varied rates were reported for raw milk, pooled from the farm, and pooled milk from the collection center.

Examining the prevalence of MRSA in milk can be used as a valuable tool for evaluating both the hygienic condition in dairy herds and the potential risks to human health exposed to antibiotic‐resistant strains. Detecting antimicrobial‐resistant bacteria in raw milk is crucial for public health due to the potential spread of these bacteria through the dairy food chain. While MRSA is relatively uncommon in food, dairy milk could be a possible source because of the widespread use of antimicrobials in treating mammary contaminations in cattle. Various factors, such as sanitation, breed, production system, management system, farm hygiene, farm size, and food safety training, were assessed through multivariable logistic regression analysis. Among these, food safety training, farm size, management, farm hygiene, and production system were significantly (*p* < .05) associated with the risk of *S. aureus* contamination.

Contrary to the findings of Regasa et al. (2019), breed and sanitation were not statistically significantly associated with the risk of *S. aureus* contamination, aligning with the reports of Mekuria et al. (2013). Food safety training emerged as highly significant for the risk of *S. aureus* contamination in dairy cows (*p* = .001). The risk of *S. aureus* contamination in dairy farm owners and milkers was 5.303 times higher than that of those who did not undergo food safety training.

The management system also showed a significant association with the risk of *S. aureus* contamination in dairy cows (*p* = .001). Dairy farms under poor management had a 7.34 times higher risk compared to those under good management, while those under medium management had a 5.1 times higher risk. The prevalence of *S. aureus* in poorly managed dairy farms was 48.7%, lower than that reported by Seyoum et al. (2018) but higher than the 10.5%  reported by Mekuria et al. (2013). This could be attributed to inadequate practices such as unsanitary conditions and the use of a single towel for teat cleaning, providing opportunities for udder and milk contamination.

The study found a significant association between the production system and *S. aureus* contamination risk in dairy cows (*p* = .032). Dairy cows under less intensive, extensive production systems had only 3.2% of the risk compared to those under semi‐intensive production systems. This finding aligns with the findings of Abunna et al. (2016), suggesting that the prevalence of *S. aureus* contamination is linked to overcrowding in semi‐intensive systems, increasing the contact rate between dairy cows and the potential for disease transmission.

Focusing on this specific topic may offer insights into improving management strategies for dairy cow production systems to reduce the risk of *S. aureus* contamination. This could lead to cost savings by decreasing the occurrence of contaminations and, consequently, lowering associated treatment expenses. Understanding the specific link between the production system and *S. aureus* prevention may empower employers to make informed decisions that benefit both the economy and the ethical treatment of animals, providing them with the best possible environment based on their needs.

The level of farm hygiene was found to be significantly linked to the risk of *S. aureus* contamination in dairy cows (*p* = .001). Dairy farms with poor hygiene were 2.88 times more likely to be at risk of *S. aureus* contamination compared to those with moderate hygiene. This observation aligns with the findings of Megersa (2015), who noted a high association between poor hygiene and *S. aureus* contamination in dairy cows. The potential cause of this correlation may be attributed to the critical role of personal hygiene among milkers and the sanitation of milking equipment, serving as crucial avenues for the transmission of *S. aureus* between cows and from barns to milk, as highlighted by Lee et al. (2012). Similarly, Elmonir et al. (2019) in Egypt detected MRSA isolates in cows, workers, and milking equipment on dairy farms, with the initial contamination occurring on the milker's hand due to inadequate hand washing, followed by subsequent contaminations from the milking bucket at the farm level.

In the current study, it was observed that 98% of workers did not wash their hands before milking. This contrasts with the findings of Elmonir et al. (2019), where approximately 47.8% of workers did not wash their hands, and also differs significantly from the situation reported in Ethiopia by Ayele et al. (2017), where all workers (100%) were reported to wash their hands. The lack of handwashing among workers, coupled with other unhygienic practices such as raw milk consumption and the absence of personal protective equipment (PPE) use, may pose a zoonotic threat to occupational health on dairy farms (Asiimwe et al., 2017). Additionally, the study suggests that the workers' lack of awareness and willingness to work while ill could contribute significantly to the dissemination of *S. aureus* contamination in the examined farms (Elmonir et al., 2019).

Examining specific practices, the study found that 56% of milking personnel practiced udder washing before milking. This contrasts with the findings of Regasa et al. (2019), where 76.5% practiced udder washing and only 8% of respondents in this study practiced udder washing. Moreover, 96% of individuals did not use separate drying towels for the udder, aligning with the findings of Abate et al. (2015), who reported that all interviewees did not use separate towels for drying the udder after washing. However, a unanimous 100% reported the practice of washing milking equipment and storage containers with detergents before milking, in contrast to Regasa et al. (2019), where 58.8% practiced this pre‐milking hygiene measure.

## 5 CONCLUSION AND RECOMMENDATIONS

This investigation unveiled the presence of *Methicillin‐resistant Staphylococcus aureus* (*MRSA*) in dairy farms found in Wolaita Sodo Town. The identification of MRSA in milk poses an elevated risk to consumers in Wolaita Sodo Town, as it may exacerbate antimicrobial resistance across various bacterial strains. Notably, all MRSA isolates demonstrated resistance to Penicillin, with *Gentamycin* and *Sulfamethoxazole‐trimethoprim* emerging as recommended drugs for addressing MRSA contaminations. Of concern, a staggering 75% of the MRSA isolates exhibited multidrug resistance to three or more classes of antibiotics, highlighting the widespread distribution of multidrug‐resistant MRSA strains originating from raw cow's milk.

Therefore, based on the above conclusions, the following points are forwarded:‐

1. Experts should regularly follow the development of drug resistance.
2. Improving personal and environmental hygiene, including milking utensils, may reduce the prevalence of *S. aureus* in milk.
3. Gentamycin and Sulfamethoxazole‐trimethoprim should be considered the drugs of choice for treatment against *S. aureus* contaminations.

## AUTHOR CONTRIBUTIONS

**Hailehizeb Tegegne:** Conceptualization (equal); data curation (equal); formal analysis (equal); funding acquisition (equal); methodology (equal); resources (equal); software (equal); supervision (equal); writing – original draft (equal); writing – review and editing (equal). **Eyoel Ejigu:** Conceptualization (supporting); data curation (supporting); resources (equal); software (equal); writing – original draft (equal); writing – review and editing (equal). **Dese Woldegiorgis:** Conceptualization (equal); data curation (equal); formal analysis (equal); funding acquisition (equal); investigation (equal); methodology (equal); supervision (equal); writing – original draft (equal). **Azeb Mengistu:** Conceptualization (equal); data curation (equal); formal analysis (equal); funding acquisition (equal); methodology (equal); resources (equal); software (equal); supervision (equal).

## FUNDING INFORMATION

There is no funding organization for this work.

## CONFLICT OF INTEREST STATEMENT

The authors declare that they have no competing interests.

## Data Availability

The data that support the findings of this study are available on request from the corresponding author. The data are not publicly available due to privacy or ethical restrictions.
